# A feasibility study of a cognitive behavioral based stress management intervention for nursing students: results, challenges, and implications for research and practice

**DOI:** 10.1186/s12912-021-00761-6

**Published:** 2022-01-21

**Authors:** Ulrik Terp, Birgitta Bisholt, Fredrik Hjärthag

**Affiliations:** 1 Department of Social and Psychological Studies, Karlstad University, Universitetsgatan 2, 651 88 Karlstad, Sweden; 2 Department of Nursing, Karlstad University, Universitetsgatan 2, 651 88 Karlstad, Sweden

**Keywords:** Feasibility, Cognitive behavioral stress management, Nursing education, Stress, Health promotion

## Abstract

**Background:**

Stress related psychological problems are growing in nursing education and constitute an essential challenge for educators. This makes research about strategies and interventions to meet these problems important. Stress management interventions need to be tested for feasibility and acceptability, before conducting large scale RCTs. The objective of our study was to assess the feasibility and acceptability of a newly developed cognitive behavioral stress management intervention for nursing students.

**Methods:**

Data were collected using a combination of standardized measurements and newly created questionnaires in combination with qualitative data. Our data included recruitment capability, sample characteristics, intervention acceptability and preliminary evaluation of participant psychological changes.

**Results:**

Findings suggested that the feasibility of conducting a full-scale evaluation was confirmed for intervention acceptability, data collection procedures, and adherence. However, difficulties relating to recruitment capability and homework were identified. All aspects taken together, the intervention was found feasible and acceptable to nursing students, and thus a potential stress management intervention for the nurse education context.

**Conclusions:**

Overall, this study provides an insight into the challenges and complexities of developing and evaluating a new brief cognitive behavioral based stress management training intervention in a nurse education setting.

**Supplementary Information:**

The online version contains supplementary material available at 10.1186/s12912-021-00761-6.

## Background

Stress-related symptoms and psychological problems increase among nursing students during their education [1, 2]. The stress has its starting points primarily in the academic and clinical context, together with stressors related to social and financial issues [3]. Unhealthy stress has dire consequences and correlates with learning difficulties, surface-oriented learning strategies, lack of motivation, relational problems, and thus negatively influences professional development and constitutes a significant challenge for nursing students to complete their education [2, 4–8]. There is also a link between stress and more severe mental health problems as anxiety and depression [9]. In addition, stress related problems are not restricted to the nursing education. Globally, there is a shortage of nurses, and there are problems related to stress within nurses work situations, and many leave their occupation [10]. Consequently, to provide students and employees with stress management skills is a key challenge for educators and future workplaces, which makes research to meet these problems important [11].

Systematic reviews have shown that stress management interventions based on a solid theoretical basis and training new behaviors have the most positive results [12–14]. Based on these principles, we developed a new cognitive behavioral therapy (CBT) - based stress management intervention for nursing students. The theoretical basis of this intervention is a mix of classical and modern CBT, which both shares the goal of helping individuals to develop adaptive behaviors [15].

### Study rationale and objectives

When planning for a full-scale RCT of an intervention, it is essential to evaluate feasibility, acceptability, and data collection procedures as a preparation [16].

This study is the third and last part of a larger study focusing on these aspects of a newly developed stress management intervention. In our first study, using quantitative analyses, preliminary effects of the intervention were examined for stress management competency, self-efficacy and self-esteem (self-reference 1). In our second study, using a qualitative analysis, participants’ perspectives of the intervention were described (self-reference 2). The goal of the present study is to identify further aspects of the intervention itself as well as elements of the implementation that need consideration and possible modifications, to answer the question “*Can it work?*”, the central question of feasibility studies [17]. According to Orsmond and Cohn [14], there are five overarching objectives of feasibility studies focusing on social and behavioral interventions in particular. These are the assessment of recruitment capability and resulting sample characteristics, intervention acceptability, procedures and measures, a preliminary evaluation of participant responses, and resources and the ability to manage the study. Three of these are related to the aim of this study and described in Table 1.

**Table 1:** Study objectives as described by domains, main questions, data and outcomes

Overarching question - Can it work?			
Domain	Main questions	Data	Study outcome
Recruitment capability	Can we recruit appropriate participants? What are the obstacles to recruitment?	1) Characteristics of the participants, drop-outs and control group 2) Recruitment obstacles and dropout rates	Primary
Intervention acceptability	Is the intervention suitable for and acceptable to participants? What are the adherence rates to the intervention? What are the challenges related to adherence?	1) CSQ-8 2) Attendance rates 3) Adherence rates	Primary
Outcome measures and preliminary evaluation	Does the intervention show promise of being successful with the intended population? Do participants provide qualitative feedback that may be indicative of the likelihood that the intervention will be successful?	1) Standardized instruments 2) Open-ended question	Secondary

Thus, the objective of this study was to investigate the feasibility of a newly developed cognitive-behavioral based stress management intervention for nursing students.

## Method

### Settings and recruitment

The study population consisted of Swedish nursing students in a three-year program (180 ECTS credits) at a medium-sized university, leading to both a professional and a Bachelor’s degree. In Sweden, nursing programs follow the directions of the government regulations regarding the length of the education and learning objectives [18, 19]. However, the university is free to decide the organization of the education and the order of courses. The clinical practice in the program accounted for approximately half of time and credits.

For this study participants were recruited by information meetings about the study, where background, aims, contents and ethical considerations were described and distributed, both orally and in writing. Interested students signed an informed consent, and were invited to join the planned intervention outside their ordinary schedule.

### Design and participants

**Fig. 1:**
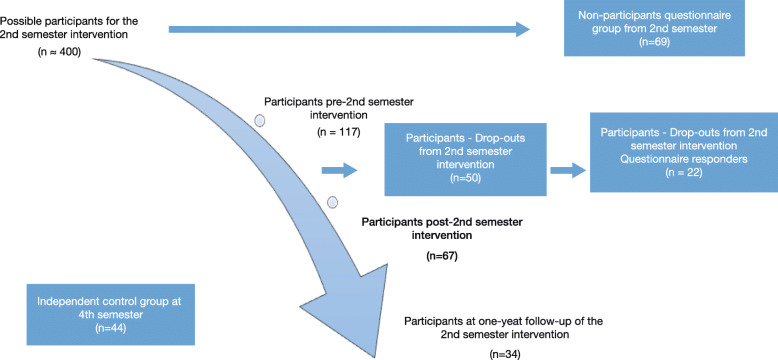
Flow diagram over study participants

At each semester over 2 years, about 400 student nurses were invited to participate in this study at their second semester. Stress during nursing education in Sweden increases mainly between semester two and four [20], similar findings are shown for other countries [21]. Thus, we found recruitment of participants from the second semester most relevant. Since recruitment capability and intervention acceptability were in focus for this study, it was important not only to follow students taking full part of the intervention, but also to follow students who chose not to participate or who dropped out from the intervention after they had started their participation. All these groups, forming the base for this study are presented in a flow diagram (Fig. 1).

Both external dropouts – operationalized as non-participating nursing students called ‘Non participants questionnaire group’ in Fig. 1 and internal dropouts –operationalized as nursing students who participated less than five times, and/or did not fill out the post intervention assessment, called ‘Participants – Drop outs’ were studied. For preliminary evaluations, a pre-, post- and one-year follow up were conducted for the intervention group. Also, an independent control group was recruited from one fourth semester of nursing students, who had not had the opportunity to take part of the intervention, for the one-year follow up comparisons. The participants taking part of the intervention followed the same syllabus as the control group.

### The intervention – a cognitive behavioral stress management training program

The first author developed the intervention built on both traditional and modern cognitive behavioral theories (Table 2) emphasizing the theoretical commonalities in goals, principles, and processes [15].

**Table 2:** Brief description of the intervention; goals, principles, and processes presented together with the theoretical and practical foundations of the intervention

Session	Main theoretical focus	Main exercise	Main change principle	Main therapeutic process	Main reference (s)
1. Introduction	A brief description of the stress concept and basic principles of CBT and the intervention as a whole	The Five-factor model	–	–	O’Donohue and Fisher [24], Kuyken, Padesky [25]
2. Emotions school	What are emotions? Why do we have emotions?	Identify the function of emotions	Attention change	Attention training	Passer and Smith [26]
3. Emotions school	Concept of acceptance and coping	What do we need to accept? What would be different if you come to peace with it?	Attention change	Acceptance/tolerance	Kåver [27]
4. The importance of thoughts	The transaction model Cognitive Traps Vulnerability vs. resilience	Decision balance	Cognitive change	Cognitive reframing	Almén [28, 29]
5. The importance of thoughts	Procrastination	Worksheet with examples	Cognitive change	Cognitive reframing Defusion	Rozental and Wennersten [30]
6. Self-compassion	What does self-compassion mean?	Create a compassionate self	Cognitive change	Cognitive reframing	Neff [31]
7. Acceptance and commitment therapy	The problem solving vs. it shows the consciousness of interpretations of different life themes Psychological flexibility	The compass of life	Context engagement Cognitive change	Behavioral exposure Defusion	Hayes, Strosahl [32]
8. Life’s balances	Review of important balances: Requirements vs. control / influence Activation vs. deactivation Effort vs. reward	Mapping your own situation Identifying barriers to change	Context engagement	Behavioral activation	Bakker, Killmer [33]
9. Effective communication	Communication behaviors	Identifying barriers to effective communication	Context engagement	Behavioral exposure Behavioral activation	Almén [28]
10. Summary					

The overarching aim with the intervention is to promote psychological health. Not primarily by reducing stress, but through increasing psychological flexibility. Psychological flexibility is defined as ”*contacting the present moment as a conscious human being, fully and without needless defense - as it is and not as it says it is – and persisting with or changing a behavior in the service of chosen values*” [22]. The ability of psychological flexibility was trained with the help of relevant theory and applied exercises. Research has shown this ability to be a central aspect of mental health [23].

Further, the intervention aimed at increasing the participants’ awareness and theoretical knowledge about the interaction between cognitions, emotions, behavior, bodily reactions and contextual factors [24, 25]. Other aims with the stress management intervention were to identify and transfer useful and trainable alternative attitudes, thoughts, emotions, and behaviors to stress-related contexts relevant to each of the participants individually. During the intervention, there was a strong emphasis on the importance of training new ways of coping with each participant’s difficulties in everyday life, thus integrating and transferring new knowledge to previously challenging situations. To achieve these goals, theory-based mini-lectures, reflective group practices, exercises, and discussions were provided ten times in two-hour sessions over a ten to 12 week period of time.

### Primary outcomes

#### Recruitment capability

Examining aspects of the recruitment process and the sample characteristics are essential in determining whether the intervention is perceived relevant to the study population [26]. These data are also important when evaluating the recruitment plan and thus in determining whether the stress management training intervention and future efficacy studies would be successful. Recruitment obstacles were assessed using a survey developed by the research team, including questions about motives for not participating in the intervention and in terms of perceived barriers and obstacles. Two different versions of the dropout survey were used to identify obstacles and barriers for participation. An eight-item version for the non-participants and an extended 14-item version for the dropout group. The response alternatives ranged from 1 = *do not agree at all*, to 4 = *totally agree* see supplemental file. This gave information relevant to the recruitment issues for each specific group.

#### Intervention acceptability

Intervention acceptability is operationalized by how the individual participant in the intervention perceive the intervention [27]. The Client Satisfaction Questionnaire (CSQ-8) was used to assess participants, overall satisfaction with the quality of the intervention [28]. The questions of the questionnaire were, for example: *To what extent has our program met your needs*, and *Have the services you received helped you to deal more effectively with your problems?* with four response alternatives from poor to excellent on a Likert scale. The scale is frequently used for these purposes and shows good reliability and validity [29]. For cut-off points, levels presented by Lally, Byrne [30] were used. Further, in the follow-up measurement, the participants answered questions about their adherence to the intervention protocol, and their attendance was assessed in each session which was used and operationalized as an additional measure of intervention acceptability. The aim was to identify factors affecting implementation ease or difficulties. These data help to answer questions about the study procedures and whether the intervention is suitable, acceptable, and appealing to participants, thus questions of importance before conducting an RCT.

### Secondary outcomes

#### Quantitative preliminary evaluation

The measures were based on a literature review and the documented psychometric properties reported in prior research with nursing students or a comparable population (Table 3).

**Table 3:** Description of the instruments used in the present study

Instrument	Response scale	Number of subscales	Number of items	Original reference	Psychometric properties
Perceived Stress Scale (PSS)	5-point Likert	–	14	Cohen, Kamarck [41]	Lee [42]
Satisfaction with life scale (SWLS)	7-point Likert	–	5	Diener, Emmons [43]	Pavot and Diener [44]
Brief Cope Scale (BCS)	4-point Likert	14	28	Carver [45]	Wong and Heriot [46]
Pure Procrastination Scale (PPS)	5-point Likert	–	12	Steel [47]	Rozental, Forsell [48]
The Hospital Anxiety and depression scale (HADS)	4-point Likert	2	14	Zigmond and Snaith [49]	Lisspers, Nygren [50]
Connor Davidson Resilience Scale (CD-RISC-S)	5-point Likert	–	25	Connor and Davidson [51]	Ahern, Kiehl [52]

Given the vast number of scales used in health research, it is important to assess if they appear to be sensitive to the effects of the intervention or if new or modified measurements are required for the intended population [17].

Descriptive data are presented in Tables 7 and 8, with comparisons of means used t-tests or one-way ANOVAs when applicable. All analyses used significance level 5% and were conducted using the IBM/SPSS software version 25.

#### Open-ended survey data

In the 1 year follow-up, an open-ended question was used in addition to the quantitative measurements, *Can you describe any changes during your last year that you attribute to the intervention* to investigate the participants’ perceptions about whether the intervention affected them. Open-ended questions (OEQs) make it possible to collect data that cannot be captured through fixed response formats [31].

From the 73 participants who followed the intervention five sessions or more, 33 participants answered the open-ended question, which was analyzed using the six phases of thematic analysis [32]. The open-ended question generated 88 statements from participants who answered the question. The qualitative analysis process was carried out with NVivo version 12, which helped the first author to manage the coding process and enable the dependability of findings. In the next step, the codes were sorted into potential themes by the first and the second author separately. After that, XX and YY read and discussed each theme with associated codes repeatedly until consensus on the theme structure was reached, and no fundamental disagreements emerged during this process. Then themes were labelled. Finally, all three authors discussed and reached consensus about the interpretation of the findings.

### Ethical approval

The study was carried out following the ethical guidelines of the latest Helsinki declaration and was approved by the Regional Ethical Board in Uppsala, Sweden (Approval number: 2014/379).

## Results

### Recruitment capability

Each semester, approximately 100 students begin their education in the nursing program. During 2015–2016, all were offered to participate in the intervention. This means that approximately 400 individuals were offered to attend over the four semesters the intervention was conducted. Totally 117 students chose to participate in the intervention, and approximately 280 chose not to participate. This gave a general average participation rate of about 29% for the current intervention.

#### Sample characteristics

First and most important for feasibility, is to answer the question ‘C*an we recruit the appropriate participants?*’ and to investigate the obstacles to recruitment. Characteristics of the sample that followed the intervention, dropouts, and the control group are provided below (Table 4).

**Table 4:** Background characteristics for intervention participants, drop-outs and for the control group

Background variables	Intervention group (*n* = 67)		Drop-outs (*n* = 50)	Control group (*n* = 44)
Sex (male/female)	7/60		6/44	3/41
Age M (SD)	26,31(6,91)		26,32(7,38)	24,70(3,43)
**Social situation**				
Married/civil partnership with children	21		11	8
Married/civil partnership without children	19		18	18
Living apart with children	1		–	–
Living apart without children	5		3	3
Single with children	–		–	1
Single without children	12		13	13
Living at home with parents or other with children	9		5	1
**Parents highest education**				
Mother				
No education	1		1	3
Elementary school	8		9	5
Upper secondary education	26		20	20
Academic education	30		20	16
Father				
No education	2		–	5
Elementary education	14		13	6
Upper secondary education	33		27	24
Academic education	18		9	8
About how many hours a week do you spend on your studies M (SD)	34,36(7,51)		31,85(9,09)	25,65(9,37)***
Do you work outside your studies (Y/N)	34/33		23/27	31/12*
Yes, about how many hours M (SD)	8,94(4,25)		10,91(5,53)	13,03(5,79)**

*The difference between the intervention group and the control group is significant according to a Chi-2 test, *p* < .05

**The difference between the intervention group and the control group is significant according to an independent t-test, *p* < .01

***The difference between the intervention group and the control group is significant according to an independent t-test, *p* < .001

The group who followed the intervention (five sessions or more) and the dropout group did not differ in any background variables measured. This shows that there are no systematic differences between those who participated in the intervention and those who chose to withdraw from participation.

However, when comparing the intervention and the control group, our data shows a difference between how much time the student spent per week on studies and vocational work. The relationship between the number of hours the students spent on average each week on studies or work looks different from semester four. In their second semester, students on average placed more time on studies in relation to work, and this relationship did not apply to the control group. Whether this is a finding relevant outside our study sample is an open question, but according to studies, this is a phenomenon relatively new to nurse education where the underlying assumption about the student is that he or she is a full or part-time student. This is an assumption with relevance for stress research [33].

In summary, our analysis of between-group differences revealed no selection bias considering sex, age, social background or educational level of the participants’ parents or in time spent on studying and working when comparing the intervention group with the intervention dropout group; thus our sample is representative of the target study population, nursing students.

#### Obstacles and dropout rates

The reasons for non-participation and for intervention dropout were examined by a survey developed by the research team (see questions in Table 5). On two occasions, the questionnaire was distributed in connection with regular teaching in a full class. Individuals present at the time of this questionnaire distribution, who participated in the intervention between one to five occasions, but who chose not to complete it, voluntarily filled out this questionnaire. Answers from this group and answers given by nursing students offered to participate in the intervention but who chose not to do so, are shown in Table 5.

**Table 5:** Obstacles for participation in the intervention for the non-participants and dropout group. High scores indicate a high level of agreement with statements

	Group Non-participants (*n* = 69)		Group Dropouts (*n* = 22)	
	*%* total agreement with statement^a^	*%* totally disagree or partly disagree with statement^b^	*%* total agreement with statement^a^	*%* totally disagree or partly disagree with statement^b^
Not interested in stress management	1	91	0	96
I do not have time because I work extra	7	73	0	61
Hobbies	12	42	9	74
Other tasks related to my studies	31	41	36	18
My family situation	3	91	9	74
The program contains home assignments	3	77	0	87
I don’t want to share things about myself with others	1	97	4	78
The program did not feel relevant when it was presented to me	11	79	0	91
Course leader’s ability to convey knowledge			0	96
The relationship between the course leader and I the student			4	74
That I did not do the home assignments for the stress management program			0	96
The relationship between me and the other students			0	100
My ability to absorb the information from the lectures			0	74
My ability to do the home assignments			0	91

a) Item score 4 = total agreement with the statement

b) Item score 1 or 2 = totally disagree or partly disagree with the statement

The table shows that the most common and most prominent reason for not completing the intervention was being busy with other tasks related to regular studies. In addition to this, time for leisure and extra work were also mentioned, but to a lesser extent as reasons for not completing the intervention. Correspondingly, those who chose not to participate in the intervention stated mainly that studies and leisure interests were the reasons for this. In addition to the fixed response options contained in the questionnaire, it was also possible to add comments to a final open question. The results of the open-ended questions showed that those who answered (*n* = 26) the question primarily mentioned long travel distances from their homes to the university as the main reason for choosing not to participate in the intervention, when there were no other scheduled activities.

### Intervention acceptability

#### CSQ-8

The participants’ satisfaction with the intervention was generally high. Most participants (81.7%) had a total score between 27 and 32 points indicating that they were very satisfied with the intervention. 18.3% scored between 21 and 26 points indicating that they were satisfied. No participants had a total score below 23 points.

The result of CSQ-8 shows that overall, the participants were satisfied with the intervention. Most of the respondents were very satisfied and would highly recommend the intervention to others. The item that received the lowest rating was about how much the intervention responded to the participants’ needs. In summary the participants expressed an overall high acceptability of the intervention.

#### Attendance rates

About half of the participants who attended the first intervention session, attended the tenth session. No specific occasions differ but overall the number of participants decreased regularly. The last group that participated, however, had a higher attendance throughout all sessions on average (Table 6).

**Table 6:** Attendance distributed over the full intervention

Session number	Group 1 Autumn 2014	Group 2 Spring 2015	Group 3 Autumn 2015	Group 4 Spring 2016
1	42	33	16	16
2	38	29	12	12
3	31	26	11	12
4	33	25	10	13
5	28	20	9	12
6	27	25	5	11
7	20	20	5	10
8	22	21	7	10
9	24	20	5	12
10	21	16	7	13

Through all the groups, there were a total of 43 participants who participated in nine or ten sessions and in total 73 participated on five occasions or more, however data from 6 of these participants were lacking at the post-intervention assessments, thus only 67 of them were considered full intervention completers in this study.

#### Adherence

At time for the post-intervention measurement, a question was asked about how the participants worked on the home assignments between each session. This measurement can be considered as one aspect of engagement with the intervention. We found that the participants did not adhere to the homework assigned each session as initially expected. From our data, it appears that a clear majority, 53 out of 73 participants, did not regularly work with the home assignments. Only six people stated that they regularly worked with home assignments.

### Outcome measures and preliminary evaluation

#### Standardized instruments

For all scales, except the pure procrastination scale as well as the perceived stress scale, there are significant differences between the pre- and post-intervention measures. For HAD-anxiety and CD-RISC, the improved results were also seen in the one-year follow-up. Regarding the Brief cope scale, it was only the sub-scales of self-distraction, self-blame as well as planning, where an improvement occurred between the first and the second measurement occasions. Regarding self-distraction and self-blame, the improved results were also seen at the one-year follow-up. The results indicate that the intervention influenced the participants positively regarding central mental health parameters (Tables 7 and 8).

**Table 7:** Comparisons between assessments M1-M3 of HAD, CD-RISC, SWLS, PPS and PSS

	M1 M (SD)	M2 M (SD)	M3 M (SD)	F	Eta^2^	Power
HAD anxiety	8.00 (3.01) ^a,b^	5.93 (1.94) ^a^	6.79 (1.90) ^b^	9.33	0.41	0.96
HAD depression	12.66 (2.38) ^a^	13.59 (1.88) ^a^	13.31 (2.47)	4.20	0.24	0.67
CD-RISC	67.88 (8.19) ^a,b^	72.44 (7.82) ^a^	72.84 (8.55) ^b^	6.64	0.37	0.87
SWLS	26.48 (5.38) ^a^	28.34 (3.95) ^a^	28.31 (3.94)	5.72	0.29	0.82
PPS	30.24 (8.89)	27.10 (7.87)	27.55 (8.27)	3.10	0.19	0.55
PSS	24.73 (7.99)	20.09 (6.61)	20.82 (5.74)	2.93	0.39	0.43

a-b Significant difference means based on Bonferroni posthoc tests. (Significance level 5%, two-tailed tests)

M1 – Measurement 1, pre-intervention

M2 – Measurement 2, post-intervention

M3 – Measurement 3, one-year follow up

**Table 8:** Comparisons between assessments M1-M3 of Brief COPE interventions

Brief COPE Dimension	M1 M (SD)	M2 M (SD)	M3 M (SD)	F	Eta^2^	Power
Self-distraction	5.86 (1.51) ^a,b^	4.59 (1.43) ^a^	5.00 (1.49) ^b^	13.54	0.50	1.00
Active coping	6.61 (0.83)	6.79 (0.88)	6.89 (0.92)	1.24	0.09	0.25
Denial	3.00 (1.24)	2.74 (0.90)	2.93 (0.96)	0.99	0.07	0.20
Use of emotional support	6.38 (1.40)	6.55 (1.12)	6.79 (0.90)	1.79	0.12	0.34
Behavioral disengagement	3.24 (1.09)	3.07 (1.13)	2.93 (0.84)	1.53	0.10	0.30
Substance use	2.14 (0.52)	2.07 (0.26)	2.10 (0.41)	0.36	0.03	0.10
Venting	5.45 (1.33)	5.38 (1.21)	5.48 (1.09)	0.10	0.01	0.06
Use of instrumental support	5.93 (1.33)	6.28 (1.16)	6.41 (1.09)	3.21	0.19	0.56
Positive reframing	6.28 (1.13)	6.31 (1.04)	6.52 (1.09)	0.72	0.05	0.16
Self-blame	5.79 (1.42) ^a,b^	4.79 (1.42) ^a^	4.90 (1.66) ^b^	13.55	0.50	1.00
Planning	6.04 (0.92) ^a^	6.57 (0.79) ^a^	6.50 (1.35)	6.87	0.35	0.89
Humor	5.38 (1.64)	5.34 (1.80)	5.24 (1.64)	0.23	0.02	0.08
Acceptance	6.50 (0.69)	6.57 (0.92)	6.64 (1.13)	0.35	0.03	0.10
Religion	2.76 (1.33)	2.97 (1.40)	3.07 (1.41)	2.45	0.15	0.45

a-b Significant difference means based on Bonferroni post-hoc tests. (Significance level 5%, two-tailed tests)

M1 – Measurement 1, pre intervention

M2 – Measurement 2, post intervention

M3 – Measurement 3, one-year follow up

In addition, analyses were made between the intervention group’s one-year follow ups (now in their fourth semester) and the fourth semester control group measurements. Independent t-tests showed that there was a difference for HAD-anxiety [*t* (68.85) = 2.07; *p* < .05], where the values were higher in the control group 7.95 (3.79) compared to the intervention group 6.53 (2.16). For PSS the values were also significantly [*t* (58) = 2.41; *p* < .05] higher in the control group 24.02 (7.99) compared to the intervention group 18.71 (6.93). For COPE-active coping, the intervention group showed a significantly [*t* (76) = 2.42; *p* < .05] higher value 6.74 (0.99) compared to the control group 6.16 (1.08). Otherwise, no other differences could be observed between the intervention group’s one-year follow up and the control group.

#### Open-ended survey data

The thematic analysis revealed three themes; ‘*Focusing on self and relating deeper to others’, ‘Changed life perspective’*, and *‘To know-how’.*

#### Focusing on the self and relating deeper to others

In the first theme, the focus is on one’s self and is primarily about self-knowledge and self-awareness. The participants described how, as a result of the intervention, they started to problematize and ask questions to themselves about demands they place on themselves and an increased acceptance of their personality.

*” It has taught me to listen to myself, even taken help to rethink / correct in situations where I could influence. I have even gained a greater understanding and knowledge of my feelings.”* (Participant 2)

Through a combination of newly acquired psychological knowledge and reflective ability, several participants described how they have been positively influenced and gained greater self-knowledge.

#### Changed life perspective

The participants described that they have learned to handle stress in a different way, which has contributed to them looking at life in a new way and with a changed life perspective. For instance, it could be a change in perspective, as to whether a challenging life situation should be a problem or not.

*” I feel that I can handle the stress in a different way, can in some way have control over it and stop stressing for example by prioritizing certain things and accepting that I cannot do everything.”* (Participant 16)

Most of the participants describe how, through a changed attitude, they have been influenced in their way of thinking and relate to the degree of control and influence one has in different situations. The participants expressed this through the question of what is possible to influence and what lies outside of control. This question contributed to the participants experiencing that they could relate differently than before to both large and small issues in life. This was seen, for example, in the view on how both study and work tasks should be handled and prioritized for several participants.

#### To know-how

The participants describe new behaviors they feel they have learned by participating in the intervention. Relatively many participants describe how they developed new concrete ways to relate to stress and problematic situations based on the theoretical models treated in the intervention. In everyday situations, participants describe that they apply ways that work.

*” I am much quicker to separate thoughts, feelings, and actions and know that one does not have to influence the other. However, I still work with it ☺”* (Participant 9)

In the one-year follow-up, several participants describe how the theoretical content of the intervention contributed to developing an understanding of stress and stress responses, which served as support and help in everyday life. Overall, data show that the participants were affected by participating in the intervention in several different ways. They expressed themselves as being calmer and better at sorting and prioritizing things according to importance and urgency. A developed ability to reflect and improve skills in stress management together with extended theoretical knowledge is described as underlying this perceived change.

## Discussion

Our study aimed to assess the feasibility of a newly developed stress management intervention, with the overarching feasibility question expressed by Orsmond and Cohn [17], “*Can it work?*”. We investigated this overarching question based on data from three domains with relevance for this question and the present study shows that the intervention is feasible in a nurse education context.

First, we investigated questions relating to recruitment capability. Our analysis revealed that of the almost 400 students, who were offered to participate in the intervention, 29% chose to participate and about 17% completed it. The most important reasons for not participating in the stress management intervention were not related to a lack of interest but were primarily due to a focus on their regular studies. Hobbies, family, and long distances to the university were other reasons that non-participant students gave when we investigated this. These factors thus constitute perceived barriers. Hammer, Grigsby [34], Hall [35], Yarbrough, Haas [36] as well as other researchers have pointed to the problem of role conflicts and the role overload that causes stress for nursing students as a result of perceived mental stress from both study, leisure and family relationships. Based on our findings, we show that this is an important relationship to address when interventions are planned within nursing education. It seems indeed essential to facilitate participation for the students by taking into account, for example, how ordinary schedules are planned so collisions or conflicts do not occur between different interests competing for the students’ time. It should be easy to choose to participate, and synchronization between regular activities and the stress management intervention creates the conditions for participation. Further, other studies discuss the problem with perceived stigma for students who seeks help with mental health issues, as well as for academic problems in the student population [37]. Stress management strategies can therefore be important for reducing mental health problems, given the part stress play in the development of more severe mental health issues [38]. We also know from research that students with the largest needs of help are the least help-seeking [39]. One possible way of reducing potential stigma for participating in interventions like the one described in this article could be to make them part of an institutions‘core curriculum, as suggested by [12].

Secondly, we addressed the domain of intervention acceptability. Most of the participants who took part in the intervention had high attendance. Of 73 participants, 43 (about 11% of 400) voluntarily participated in nine or ten sessions. This is particularly interesting considering the general problems in attendance within nursing education when parts are not mandatory [40].

Considering homework adherence, one central aspect of CBT, we found that a majority did not explicitly express that they did this part of the intervention regularly. This is an interesting finding given their expressed high overall satisfaction with the intervention. There are several possible explanations for this phenomenon. It is possible that the group per se is one important aspect of the results. This is well known from research in group therapy and thus potentially relevant in our setting as well [41].

We measured acceptability with three different measurements: CSQ-8, attendance rates, and adherence. Taken together, these measures showed that participants were satisfied with the intervention. Based on our findings, we find our newly developed intervention feasible in the nurse education setting.

Finally, we examined questions relating to the outcome measurements and the preliminary findings of the intervention. We found positive results regarding the majority of the measurements used for the intervention group. The comparison between the intervention and control group showed differences in anxiety, stress, and active coping. These results were also strengthened by both the qualitative and quantitative one-year follow-up for the intervention group. From our open-ended survey data, it appears that most of the participants were affected in several different ways by participation; they felt calmer and better equipped to face stressful situations. The participants also reported that they developed their existing approaches and strategies for private, study, and work-related areas. Our intervention is based on a theoretical mix of both classic and modern CBT. By blending theoretical views and exercises, the participants were trained to become more aware of the interplay between thoughts, feelings, and behaviors, thus gaining tools and insights that could help them both analyze and guide behavior.

However, it is possible that there are reasons to use instruments with greater sensitivity. In a previous study, we used a self-developed instrument to measure stress management competence, which needs to be evaluated psychometrically. This study also reviewed General Self-efficacy with the instrument General Self-efficacy Scale (GSE) as well as Self-esteem with the instrument Rosenberg Self-Esteem Scale (RSES). These instruments showed sensitivity to the intervention in question (self reference). Given the focus of the intervention, there are further possible psychological changes that would be relevant to examine. This applies primarily to psychological flexibility, a measurement not initially identified as crucial, but might be worth to consider for future studies.

There is extensive research on stress and stress-related problems among students in higher education in general and regarding nursing education globally. However, there is much less research on various interventions aimed at investigating this problem, and to the best of our knowledge, this will be the first study to look at the feasibility of delivering and evaluating a CBT-based stress management training intervention for nursing students. It is also, to our knowledge, the first study to examine the reasons not to participate in a stress management intervention. This is a research contribution taking into consideration the magnitude of the global stress-related problems, such as mental health issues and learning difficulties related to stress. Our findings could be of relevance to researchers, nurse educators as well as educators in a broader sense given that stress-related problems are not limited to nursing education, but concern higher education and those who work there in general [42].

### Challenges to address in future studies

Our study also demonstrates that there are challenges to consider when implementing a CBT-based stress management intervention. One such aspect of the implementation that is crucial to consider concerns homework. Homework and skill practice is attributed great importance within CBT because the work of learning new ways of thinking, interpreting, and behaving mainly take place between each session [43]. Even in clinical research, this is an area identified as an Achilles heel [44–46]. Based on our findings, we consider this a significant challenge to address in future studies. The primary responsibility for this challenge lies with the group leader and his/her leadership, and interpersonal approach can be decisive for the outcome [47]. The degree of warmth, openness, and empathy, together with the ability to understand and react to different aspects of group processes has been shown to be important for outcomes. Training in these respects should, therefore, be a key element in the education to lead interventions [41]. Based on our findings with the challenge of addressing the homework effectively, we suggest that in future studies, the facilitators should be working in pairs.

Further, our data showed that for many, long distances to the university were important reasons not to participate in the intervention. An opportunity to face this problem could be to supplement the intervention with a digital counterpart or a hybrid form that would allow more people to participate. Also, participating digitally via internet-based alternatives has the equivalent results for several psychological problem areas [48].

In a wider perspective, it could also be relevant with multi-centered studies and international collaborations, to investigate these problems globally. Longitudinal studies of nursing students as they later take on RN roles, could also add relevant new knowledge to this research area.

### Strengths and limitations

There are several strengths in our study. To the best of our knowledge, few other studies follow up theoretically well-supported ten session interventions using 12 months for a follow-up time. Turner and McCarthy [49] have previously requested studies like this. We have data regarding both barriers to participation and for challenges directly linked to the implementation of the intervention itself. We have specifically examined the participants’ perspectives using both quantitative and qualitative data, which gives strength to the results. This is also requested in the literature [26].

However, our study also has some limitations. There are some aspects of the concept of feasibility and acceptability that we have not addressed. Our study focuses mainly on aspects related to recruitment and acceptability. Clinical outcomes fall into the background, and other important aspects such as integration and health economy are left out of the focus of the study, which is a shortcoming in our study. Further, the study design is weak to detect possible effects when evaluating the secondary outcomes and the limited sample should be considered when interpreting our findings. As in many studies, there is a risk of a positive selection bias, where participants that are more interested adhere and provide data, while less interested participants drop out.

## Conclusions

The main purpose of this study was to examine the feasibility of a newly developed stress management intervention for nursing students.

Regarding the first domain, recruitment capability, there were no significant differences in the investigated background variables between the intervention participants and non-participants. Further, approximately 29% of the students who received the offer to participate choose to do so. The main obstacles expressed by non-participants were prioritizing their regular studies.

The second domain concerned intervention acceptability operationalized with the client satisfaction scale, attendance and adherence rates. In summary, the participants’ expressed high intervention satisfaction. Based on our findings our study revealed lack of adherence to a specific aspect, homework, of the intervention and recommendation for a dual leadership model were discussed as one way of addressing this problem in future studies.

The third domain investigated regarded outcome measures and an overall preliminary evaluation. Our examination of both quantitative and qualitative data from the participants of the intervention proposed that the intervention, all domains investigated taken together, has the potential of being successful with nursing students. The standardized measures used in our study worked well, but we suggest that future studies measure psychological flexibility, being a more relevant outcome measure for a modern CBT-based intervention.

One can conclude that the intervention may constitute an example of the type of intervention that is requested in the literature by both clinicians and researchers. It has been carried out with relatively simple means, is theoretically well substantiated, easily accessible to the participants, has high acceptability and demonstrated both short-term, but also preliminary, long-term positive results for the participants.

## Supplementary Information

**Additional file 1.** Follow-up of the CBT-based stress management project for nursing students.

## Data Availability

The datasets and analyses for the current study are not publicly available. However, the materials could be available from the corresponding author upon reasonable request.

## Abbreviations

CBT: Cognitive behavioral therapy
CD-RISC: The Connor-Davidson resilience scale
CSQ-8: The client satisfaction questionnaire
GSE: General self-efficacy
HAD: Hospital anxiety and depression scale
RCT: Randomized controlled trial
