# Supplementary material for: A feasibility study of a cognitive behavioral based stress management intervention for nursing students: results, challenges, and implications for research and practice
Source: BMC Nurs. 2022 Jan 21;21:30. doi: 10.1186/s12912-021-00761-6 (PMC8780998; doi:10.1186/s12912-021-00761-6)
Supplement: Supplementary file 1 — Additional file 1. Follow-up of the CBT-based stress management project for nursing students. [file 12912_2021_761_MOESM1_ESM.docx]

Follow-up of the CBT-based stress management project for nursing students

To improve and follow up the stress management program that was implemented in autumn 2014 we would be grateful if you answered the following questionnaire about questions related to participation or that you chose not to participate in the project. Your answers are important for studies related to this type of program. Thank you for helping us!

If you have chosen NOT to participate in the program at all, just answer questions 1-9 below (page 1).
For those of you who participated between 1-5 times, we ask you to answer the questions on the next page (page 2).

Female □ Male □

Age ____years

|  |  | Disagree | Partly disagree | Partly agree | Totally agree |
| --- | --- | --- | --- | --- | --- |
|  | I have not participated in the program due to: |  |  |  |  |
| 1. | I'm not interested in the subject of stress management |  |  |  |  |
| 2. | I do not have time because I work extra |  |  |  |  |
| 3. | Hobbies |  |  |  |  |
| 4. | Other tasks related to my studies |  |  |  |  |
| 5. | My family situation |  |  |  |  |
| 6. | The program contained home assignments |  |  |  |  |
| 7. | That I do not want to share things about myself with others |  |  |  |  |
| 8. | The program did not feel relevant to me when it was presented to me |  |  |  |  |

9. Other reasons: ___________________________________________________________________

For those of you who participated between 1-5 times, we ask you to answer the questions on the next page (page 2).

Thank you for your participation!

1. For those of you who participated between 1-5 times, we ask you to answer the questions below (page 2).

Female □ Male □

Age ____years

|  | Disagree | Partly disagree | Partly agree | Totally agree |
| --- | --- | --- | --- | --- |
| I have not participated in the program due to: |  |  |  |  |
| 1. I'm not interested in the subject of stress management |  |  |  |  |
| 1. I do not have time because I work extra |  |  |  |  |
| 1. Hobbies |  |  |  |  |
| 1. Other tasks related to my studies |  |  |  |  |
| 1. My family situation |  |  |  |  |
| 1. The program contained home assignments |  |  |  |  |
| 1. That I do not want to share things about myself with others |  |  |  |  |
| 1. The program did not feel relevant to me when it was presented to me |  |  |  |  |
| 1. The course leader's ability to convey knowledge |  |  |  |  |
| 1. The relationship between the course leader and me as a student |  |  |  |  |
| 1. That I did not do the homework for the stress management program |  |  |  |  |
| 1. The relationship between me and the other students |  |  |  |  |
| 1. My ability to absorb the information from the lectures |  |  |  |  |
| 1. My ability to complete the home assignments |  |  |  |  |

1. Other reasons:

_____________________________________________________________________

Thank you for your participation!
